# Exploration of Zoo felids in North-East China for the prevalence and molecular identification of *Cryptosporidium* spp.

**DOI:** 10.7717/peerj.11819

**Published:** 2021-08-19

**Authors:** Shakeel Hussain, Syed Mohsin Bukhari, Lixin Wang, Nimra Khalid, Zhijun Hou

**Affiliations:** 1 College of Wildlife and Protected Area, Northeast Forestry University, Harbin, China; 2 Department of Wildlife and Ecology, University of Veterinary and Animal Sciences, Lahore, Pakistan; 3 Key Laboratory of Wildlife Conservation, China State Forestry Administration, Harbin, China

**Keywords:** Cryptosporidium, Zoo felids, 18S rRNA gene, Prevalence, Phylogenetic

## Abstract

*Cryptosporidium* spp. is a protozoan having the potential to cause zoonosis in humans and animals. Despite the zoonotic importance of this protozoan parasite, limited data are available about its prevalence in zoo felids in North-Eastern China. Hence, the current study was designed to determine the occurrence and molecular characterization of *Cryptosporidium* spp. from the fecal samples of captive zoo felids. Fecal samples (*N* = 244) were collected from different felids from five different zoos of North-Eastern China. 18S rRNA gene was amplified from the genomic DNA using species specific primers in nested polymerase chain reaction (nPCR) and *Cryptosporidium parvum* and *Cryptosporidium* spp. was found. The overall prevalence of *Cryptosporidium* was 9.43% (23/244). The 18S rRNA gene similarity analysis showed that 6 *Cryptosporidium* isolates were *Cryptosporidium parvum* and the remaining 17 *Cryptosporidium* isolates were resembling to a *Cryptosporidium* spp., which is similar to *Cryptosporidium* NEV10. Phylogenetic tree was constructed based on 18S rRNA of *Cryptosporidium* spp. The similarity of *Cryptosporidium parvum* was with its other isolates in China, India, Iran, Iraq, Turkey, Czech Republic, Spain and USA while *Cryptosporidium* NEV10 alike had a close relationship with Turkish isolates. In conclusion, *Cryptosporidium* was prevailing in feline animals of China zoo and zoo officials are directed to consider their control policy as it can be a cause of zoonosis.

## Introduction

*Cryptosporidium* is an important enteric parasite/protozoan in human beings, pets, domesticated and wild animals and is among the top four causes of moderate-to-severe diarrheal disease in young children in developing countries (Cui et al., 2014; Liu et al., 2014; Ryan & Hijjawi, 2015). *Cryptosporidium* spp. causes a diarrhea leading to fatal illness in the immunocompromised host. Fecal contaminated water and food is usually responsible of the transmission of *Cryptosporidium* (Zahedi et al., 2016).

Genus *Cryptosporidium* has 38 species and about 70 genotypes (Feng, Ryan & Xiao, 2018). Among these 38 species, 20 have been identified in humans but most of them are caused by *C. hominis* and *C. parvum* (Holubová et al., 2016; Kváč et al., 2016; Li et al., 2015a; Ryan, Fayer & Xiao, 2014; Ryan et al., 2015) which are the significant source of human cryptosporidiosis. Additionally, some other *Cryptosporidium* species and genotypes have also been described in certain reports. It includes *C. andersoni*, *C. fayeri*, *C. cuniculus*, *C. ubiquitum*, *C. suis*, *C. muris*, *C. canis*, *C. felis*, and *C. meleagridis* species and five genotypes named as pig genotype II, monkey genotype, horse genotype, chipmunk I genotype and skunk genotype (Cama et al., 2003; Sulaiman et al., 2005; Feng et al., 2007; Robinson, Elwin & Chalmers, 2008; Fayer, 2010; Waldron, Cheung-Kwok-Sang & Power, 2010; Xiao, 2010). These animals have the capacity to contaminate the water resources with different waterborne zoonotic species of organisms like *Cryptosporidium* (Zahedi et al., 2016). Alike world countries, China is focusing on the epidemiological survey of *Cryptosporidium* infection in humans, environmental water, and domesticated and wild animals (Yin et al., 2013).

*Cryptosporidium felis* is the most common protozoan in the domestic cats first reported by Iseki (1979). Afterward, highly zoonotic *Cryptosporidium parvum* (Sargent et al., 1998) and *Cryptosporidium muris* (Pavlasek & Ryan, 2007) were also reported in the same host. Recently, molecular epidemiological investigations of cryptosporidiosis in captive wild felines have gained more attention.

Previously, the prevalence of *Cryptosporidium* has been studied in different captive wild felids (Yin et al., 2013; Gómez et al., 1996; Fagiolini et al., 2010; Li et al., 2015b; Lukášová et al., 2018; Alves et al., 2005; Mateo et al., 2017; Ziegler et al., 2007; Carver et al., 2012; Holsback et al., 2013). South China tiger (*Panthera tigris tigris*), Black Panther (*Panthera pardus*) and Manul (*Felis manul*) are the notable epidemiological studies on wild felids in China (Yin et al., 2013; Li et al., 2015b).

The present study was designed to find out the prevalence and molecular identification of *Cryptosporidium* spp. in zoos including Siberian tiger (*Panthera tigris altaica*), Bengal tiger (*Panthera tigris tigris*), white tiger (*Panthera tigris*), Jaguar (*Panthera onca*), white lion (*Panthera leo*), African lion (*Panthera leo leo*), cheetah (*Acinonyx jubatus*), caracal (*Caracal caracal*), African serval (*Leptailurus serval*) and bobcat (*Lynx rufus*) from the different zoos of Beijing, Dalian, Longyan and Harbin.

## Materials & Methods

### Sample collection

**Figure 1:**
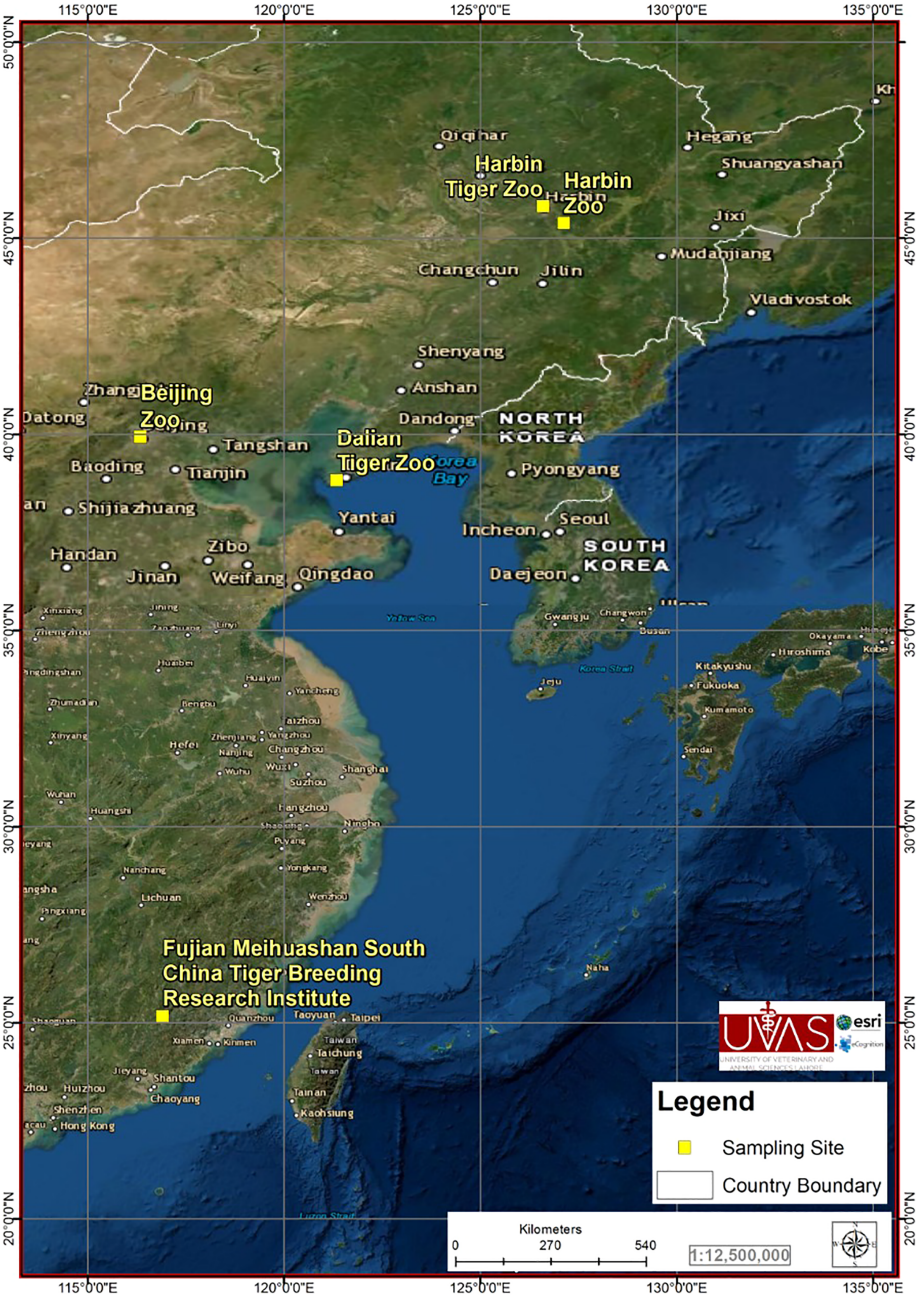
City location in which the samples were collected.

From May 2018 to September 2019, 244 samples (20–30 g each) were collected from Beijing zoo (38), Dalian tiger zoo (38), Fujian Meihuashan South China tiger Breeding Research Institute (10), Harbin zoo (56) and Harbin tiger zoo (102) of China (Fig. 1). The zoos selection criterion was the abundance of feline species in zoos, zoos with high number of feline species were included in the study. All the collected 244 samples were from the apparently healthy hosts of Siberian tiger (*Panthera tigris altaica*) (146), African lion (*Panthera leo*) (8), Bengal tiger (*Panthera tigris tigris*) (8), Caracal (*Caracal caracal*) (18), Cheetah (*Acinonyx jubatus*) (16), Jaguar (*Panthera onca* ) (11), Lynx (*Lynx canadensis*) (5), White lion (*Panthera leo*) (13), White tiger (*Panthera tigris tigris*) (8) and African serval (*Leptailurus serval*) (11). Estimated relative abundance of the sampled species was 60%. We observed maximum individual animals at different sites available within zoos for defection and collected freshly deposited fecal samples from the ground of the cages (fecal samples were collected within half hour after deposition). Fecal samples were mixed with 5% freshly prepared solution of potassium dichromate (Sigma–Aldrich, St. Louis, MO, USA) in 1:3 mass to volume ratio and stored at 4 °C.

### Oocyst isolation

Sheather’s solution was used to isolate oocysts through the discontinuous sucrose gradients. This solution contained 320 ml water, 500 g sucrose, 9 ml phenol and diluted with distilled water and supplemented with Tween-80 (1%). The 1:2 solution and 1:4 solution have different specific gravity. In 50 ml centrifuge tubes, 15 ml of the 1:4 solutions were layered over 15 ml of the 1:2 solutions. A 10 ml aliquot of the sieved feces was layered at the top and centrifuged at 1,000×*g* for 25 min. The layer between the 1:2 solutions and 1:4 solutions was aspirated very gently and put into a new 50 ml centrifuge tube. Distilled water was supplemented up to 45 ml and centrifuged at 3,000×*g* rpm for 10 min. Discarded the supernatant and dissolved the sediment (oocysts) with 500 µL distilled water (Arrowood & Sterling, 1987).

### DNA extraction

Genomic DNA was extracted from oocysts using QIAamp DNA Mini Stool Kit (Qiagen, Hilden, Germany) with slight modification in manufacturer instruction. The modification adjusted were lysis temperature at 80 °C and elution buffer to 80 ml to increase the DNA concentration. The genomic DNA was stored at −20 °C until further usage.

### Nested PCR analysis of the 18S rRNA

A two-step nested PCR was optimized for diagnosis of *Cryptosporidium* spp. Forward primer (F1) 5′-TTCTAGAGCTAATACATGCG-3′ and reverse primer (R1) 5′-CCCTAATCCTTCGAAACAGGA-3′ was used in the first step of PCR to amplify a product of 1,325 bp. A product of 830 bp was amplified in the second PCR step using forward primer (F2) 5′-GGAAGGGTTGTATTTATTAGATAAAG-3′ and reverse primer (R2) 5′-AAGGAGTAAGGAACAACCTCCA-3′ (Xiao et al., 1999). Totals volume 25 μl containing 2.5 μl PCR buffer, 2 μl Magnesium Chloride, 2 μl dNTP, 1 μM of each primer, 1 μl of Taq DNA polymerase and 3 μl of DNA template. The optimized thermocyclic conditions were initial denaturation at 94 °C for 5 min followed by 30 cycles (94 °C for 30 s, 54 °C for 30 s and 72 °C for 90 s) and final extension at 72 °C for 7 min. The PCR products were run on 1% gel and purified with Gel Extraction Kit-200 (OMEGA, China). In second step, PCR products of 1st step were used as templates. In it, initial denaturation and final extension remained the same during but the 30 cycles consisted of 94 °C for 30 s, 56 °C for 30 s, and 72 °C for 60 s.

### DNA sequencing and species/genotypes identification

The nPCR products were run on 1% gel and purified by Gel Extraction Kit-200 (OMEGA, China) and sent for sequencing to Comate Biosciences Co., Ltd. (Changchun, China). Successfully sequenced data was run on NCBI for similarity analysis using BLAST.

### Phylogenetic analysis

MegAlign (Version 4.0 DNA Star, Madison, USA) was used to align the obtained sequences for genotyping. These sequences were submitted to GenBank (accession numbers can be seen in data availability). The phylogenetic tree was constructed using the Maximum-Likelihood method on MEGA version 6.0.

### Statistical analysis

Descriptive frequencies of *Cryptosporidium* were shown in percentages by dividing the positive *Cryptosporidium* sample to the total number of samples. Chi square test of independence at significance level *α* = 0.05 was used to determine the association of *Cryptosporidium* prevalence with the sampling site. Sampling sites (zoos) and prevalence (positive and negative sample) were handled as nominal variable and Chi square test of independence was applied using IBM SPSS Statistics 20.

## Results

### *Cryptotosporidium* prevalence in Feline

A total of 244 samples were used for nested-PCR and 23 (9.43%) specimens were found positive for *Cryptosporidium* (Table 1). Among these 23 positives, 22 samples were from Harbin zoo and one from Beijing zoo while Dalian tiger zoo, Fujian Meihuashan South China tiger Breeding Research Institute and Harbin tiger zoo were negative for *Cryptosporidium*. The prevalence varied from 0.0 to 39.3% in different zoos. Prevalence of *Cryptosporidium* spp. was 8.22% (12/146) in Siberian tiger, 12.5% (1/8) in Bengal tiger, 37.5% (3/8) in white tiger, 15.38% (2/13) in white lion, 40% (2/5) in lynx and 25% (2/8) in African lion while it was not prevalent in caracal (18), cheetah (16), jaguar (11) and Serval (11) (Table 2).

**Table 1:** *Cryptosporidium* spp. genotypes in zoo field samples in China.

Sr. no.	Sampling site	No. of samples	*Cryptosporidium parvum*	*Cryptosporidium* sp. NEV10 Alike	Total positive tested	Chi square test of independence
1	Beijing Zoo	38	01	--	01	*X*^*2*^ (4) = 76.15 *p* < 0.01 *φc* = 0.56 *n* = 244
2	Harbin Zoo	56	05	17	22	
3	Fujian Meihuashan South China tiger Breeding Research Institute	10	--	--	--	
4	Dalian tiger zoo	38	--	--	--	
5	Harbin tiger zoo	102	--	--	--	
	Total	244	06	17	23	

**Note:**

*“φc”* denoting Cramér’s V.

**Table 2:** Host specie wise *Cryptosporidium* spp. genotypes in zoo field samples in China.

Sr. no.	Host	No. of samples	*Cryptosporidium Parvum*	*Cryptosporidium* sp. NEV10 alike	Total positive tested	Negative samples	Percentage
1	Siberian Tiger	146	03	09	12	134	8.22
2	Bengal Tiger	08	01	–	01	07	12.5
3	White Tiger	08	01	02	03	05	37.5
4	White Lion	13	01	02	03	10	23.1
5	Caracal	18	–	–	–	18	–
6	Cheetah	16	–	–	–	16	–
7	Lynx	05	–	02	02	03	40
8	Jaguar	11	–	–	–	11	–
9	African Lion	08	–	02	02	06	25
10	Sarval	11	–	–	–	11	–
	Total	244	06	17	23	221	9.43

A chi-square test of independence was performed to examine the relationship between zoo and *Cryptosporidium* spp. prevalence. The relationship between these two was significant *X*^*2*^ (4, *N* = 244) = 76.15, *p* = 0.00. The effect size for these finding, Cramer’s V, was very strong, 0.56. As can be seen in Table 1, from the total positive sample tested in this study 95.65% of the positive sample were from the Harbin Zoo, it means feline species of Harbin zoo has significantly higher prevalence than the other zoo.

### *Cryptotosporidium* spp./genotypes

**Figure 2:**
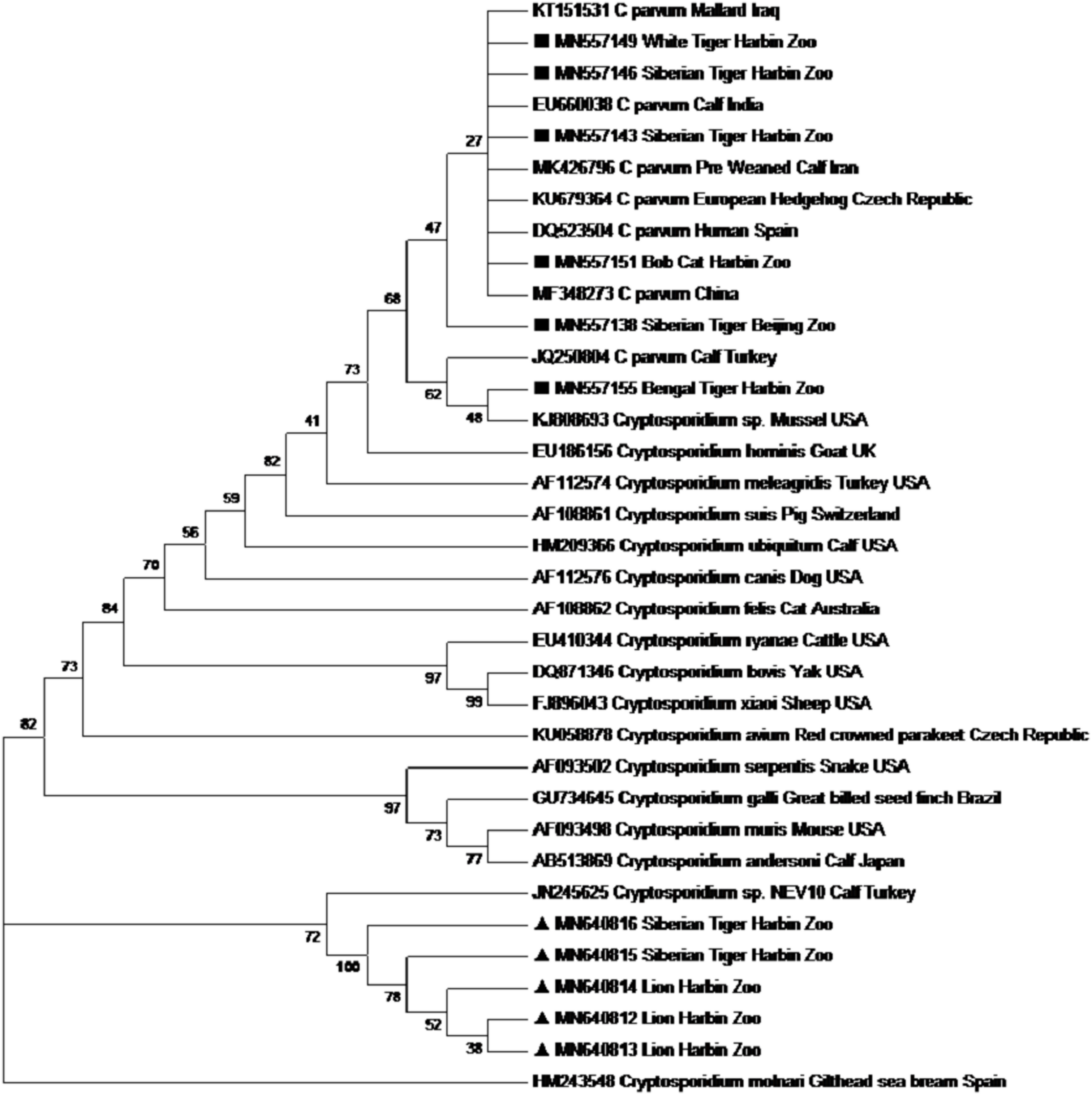
Phylogenetic tree showing the sequences of the present study, ▪ for *Cryptosporidium* *parvum* and ▴ for *Cryptosporidium* sp. NEV10 alike, remaining sequences are from NCBI GenBank data base.

Similarity analysis of the 18S rRNA gene showed that 6 out of 23 were *Cryptosporidium parvum* and the remaining 17 isolates resembled to *Cryptosporidium* spp. NEV10 (JN245625). For explaining the phylogenetic relationship, a tree was constructed based on 18S rRNA using the maximum likelihood method (Fig. 2). *Cryptosporidium* with accession numbers MN640812, MN640813, MN640814, MN640815 and MN640816 was identical to the reference sequence (accession no. JN245625, *Cryptosporidium*NEV10) that was isolated from a diarrheic calf in Turkey. Six Isolates with accession numbers MN557138, MN557143, MN557146, MN557149, MN557151 and MN557155 were identical to that of *Cryptosporidium parvum*.

## Discussion

In present study, *Cryptosporidium* was prevalent in 9.43% (23/244) in five captive felids of Chinese Zoos. Lynx was having the highest prevalence rate for *Cryptosporidium* 40% (2/5) and lowest prevalence was observed in Siberian Tiger 8.22% (12/146). While, in term of locations the prevalence ranges from 0% to 39.3% in five different zoos. Harbin zoo was having the highest prevalence rate of *Cryptosporidium*.

Wildlife plays a crucial role in spreading various pathogens to animals (domestic, Pet, free-living) and humans (Thompson, Kutz & Smith, 2009). Various studies on epidemiological surveys have been conducted around the world considering their importance to public and veterinary health (Ryan, Fayer & Xiao, 2014; Feng et al., 2007). Although few studies have been conducted for the detection of *Cryptosporidium* in different wild and captive animals. In the case of zoo felids, only manul (*Felis manul*) was found infected with *Cryptosporidium felis* in Zhengzhou zoo of China (Li et al., 2015b) and Bobcat (*Lynx rufus*) with *Cryptosporidium parvum* in the USA (Carver et al., 2012). The present study will benefit our understanding on the prevalence of *Cryptosporidium* in zoo Felids.

The *Cryptosporidium* prevalence in the present study is 9.43% (23/244) in five captive felids of Chinese Zoos it coincides with the results reported previously in wild red fox in Spain 8% (7/87) and in animal sources (caged dogs, kennel cats, captive animals) in China 7% (6/84) (Yin et al., 2013; Mateo et al., 2017). Higher proportion of *Cryptosporidium* prevalence has been reported previously in wild and domestic mammals in South Africa (14%, 8/56) and in free-living wild felids in Brasil (15%, 2/13) (Lukášová et al., 2018; Holsback et al., 2013). Fewer studies also revealed lower rates of *Cryptosporidium* in wild and captive reptiles in USA 3% (15/528) and in captive Asiatic black bears (*Ursus thibatenus*) in China 2.4% (4/218) (Upton et al., 1989; Wang et al., 2020). In Italy, parasitic investigation in mammals of two zoological gardens reported the prevalence of *Cryptosporidium* in carnivores (10%), primates (66.7%) and artiodactyls (25%) (Fagiolini et al., 2010). In Lisbon Zoo, *Cryptosporidium* was found in one Indian star tortoise (*Geochelone elegans*), one Prairie bison (*Bison bison bison*) and one black wildebeest (*Connochaetes gnou*) (Alves et al., 2005). While in a study held in 1999, *Cryptosporidium* was found in 18 Artiodactyla, 14 Primate, 2 Perissodactyla, and 1 Proboscidea species of Barcelona zoo (Gomez et al., 2000). In wildlife populations within a watershed landscape, the prevalence rate of *Cryptosporidium* was 5% (312/6227) in southeastern New York State (Ziegler et al., 2007).

Current results of highest prevalence rate 39.3% in the Harbin zoo can be compared with the result of a previous study conducted in Zhengzhou zoo where prevalence rate was very low 1.5% (3/203) (Li et al., 2015b). Same in the Lisbon zoo, only three out of 274 fecal samples from mammals and reptiles were found positive of *Cryptosporidium* (Alves et al., 2005) while another study revealed 3.6% (14/388) in ruminants of the same zoo (Delgado et al., 2003). In 1996, Only six out of 51 mammal species were found to be infected from *Cryptosporidium* in Barcelona zoo (Gómez et al., 1996).

In our study, *Cryptosporidium parvum* and *Cryptosporidium* spp. NEV10 alike were identified in zoo felids. *Cryptosporidium parvum* has a zoonotic potential and previously found in preweaned calves and humans in China (Qi et al., 2015; Wang et al., 2013). Only from Chile, a sequence of *Cryptosporidium parvum* (accession no. GQ865534) in Bengal tiger has been submitted to NCBI GenBank before the findings of current study. To the best of our knowledge, manul (*Felis manul*) was infected from *Cryptosporidium felis* in Zhengzhou zoo which was the only published report of any zoo felid having cryptosporidiosis in China (Li et al., 2015b).

Another *Cryptosporidium* spp. was detected in the present study which showed up to 100% identity with the published sequence under the accession number JN245625 (*Cryptosporidium* spp. NEV10). Previously, *Cryptosporidium* spp. NEV10 was only found in diarrheic calf in Turkey. Because of the limited knowledge of this species, its significance about the public-health and whether zoo felids from China are the new host of this species is unclear. The prevalence of zoonotic *Cryptosporidium parvum* and *Cryptosporidium* spp. NEV10 alike might have link with the contaminated feed and water as zoo felids were fed with chicken and beef. Findings of this study demonstrated that, felid species were the suitable hosts for very common zoonotic *Cryptosporidium* sp. (C. *parvum*) and *Cryptosporidium* spp. NEV10 alike.

However, during this study all the members of a felid species available at zoo were not accessed, as animal freely roam in the garden of the zoo.

## Conclusions

The study under taken showed that first evidence of molecular identification of *Cryptosporidium parvum* in the zoo felids of China and those hosts may act as carrier of zoonoses by shedding the oocysts of *Cryptosporidium parvum*. The identification of an unknown *Cryptosporidium* spp. which has a close relationship with *Cryptosporidium* NEV10 from Turkey directs for further investigation as it was detected from diarrheic calf but now it was found from zoo felids. This study directs the attention of animal welfare zoo officials to take control measures such as proper housing and management to provide environment that allow animal to grow, mature, reproduce and minimizes the effect of disease due to parasites.

## Additional Information and Declarations

### Field Study Permissions

The following information was supplied relating to field study approvals (*i.e*., approving body and any reference numbers): We collected the samples from zoo and were provided verbal consent by the manager of each zoo. The manager names are: Xiuyun Like from Harbin Zoo Dan Liu from Harbin Tiger Zoo of China Chenling Zhang from Beijing Zoo Wenyuan Fu from Fujian Meihuashan South China Tiger Breeding Research Institute Fengcheng Hou from Dalian Tiger zoo

### DNA Deposition

The following information was supplied regarding the deposition of DNA sequences: The *Cryptosporidium parvum* 18S rRNA gene sequences are available at NCBI GenBank: MN557138, MN557143, MN557146, MN557149, MN557151, MN557155; Cryptosporidium spp. NEV10 alike: MN640812–MN640816.

### Data Availability

The following information was supplied regarding data availability: The data is available at NCBI GenBank: MN557138, MN557143, MN557146, MN557149, MN557151, MN557155 and MN640812–MN640816.
